# Preliminary fMRI findings concerning the influence of 5‐HTP on food selection

**DOI:** 10.1002/brb3.594

**Published:** 2016-10-28

**Authors:** Stephanos Ioannou, Adrian L. Williams

**Affiliations:** ^1^ Department of PhysiologyCollege of MedicineAlfaisal UniversityRiyadhSaudi Arabia; ^2^ Department of Life Sciences & Centre for Cognitive NeuroscienceBrunel University LondonUxbridgeMiddlesexUK

**Keywords:** 5‐HTP, anorexia, food consumption, functional magnetic resonance imaging, obesity, serotonin

## Abstract

**Objective:**

This functional magnetic resonance imaging study was designed to observe how physiological brain states can alter food preferences. A primary goal was to observe food‐sensitive regions and moreover examine whether 5‐HTP intake would activate areas which have been associated with appetite suppression, anorexia, satiety, and weight loss.

**Methods and Procedure:**

Fourteen healthy male and female participants took part in the study, of which half of them received the supplement 5‐HTP and the rest vitamin C (control) on an empty stomach. During the scanning session, they passively observed food (high calories, proteins, carbohydrates) and nonfood movie stimuli.

**Results:**

Within the 5‐HTP group, a comparison of food and nonfood stimuli showed significant responses that included the limbic system, the basal ganglia, and the prefrontal, temporal, and parietal cortices. For the vitamin C group, activity was mainly located in temporal and occipital regions. Compared to the vitamin C group, the 5‐HTP group in response to food showed increased activation on the VMPFC, the DLPFC, limbic, and temporal regions. For the 5‐HTP group, activity in response to food high in protein content compared to food high in calories and carbohydrates was located in the limbic system and the right caudomedial OFC, whereas for the vitamin C group, activity was mainly located at the inferior parietal lobes, the anterior cingulate gyri, and the left ventrolateral OFC. Greater responses to carbohydrates and high calorie stimuli in the vitamin C group were located at the right temporal gyrus, the occipital gyrus, the right VLPFC, whereas for the 5‐HTP group, activity was observed at the left VMPFC, the parahippocampal gyrus bilaterally, the occipital lobe, and middle temporal gyri.

**Discussion:**

In line with the hypotheses, 5‐HTP triggered cortical responses associated with healthy body weight as well as cerebral preferences for protein‐rich stimuli. The brain's activity is altered by macronutrients rich or deprived in the body. By reading the organisms physiological states and combining them with memory experiences, it constructs behavioral strategies steering an individual toward or in opposition to a particular food.

## 1 Introduction

The view that desire for food is elicited to satisfy nutritional needs is well established (Pelchat, Johnson, Chan, Valdez, & Ragland, 2004). In support of this argument Weingarten and Elston (1991) postulated that cravings arise due to nutrient or caloric deficits. However, there are occasions where craving for food is not elicited in response to physiological needs. In such occasions, the desire to eat can lead to obesity, eating disorders, and noncompliance with dietary restrictions (Berthoud & Morrison, 2008; Cangiano et al., 1992; Levin & Routh, 1996; Ouwehand & Papies, 2010). Although researchers find it extremely difficult to illustrate a relation between nutritional needs and food cravings, other studies in the “food arena” exploring the relationship between the neurotransmitter serotonin (5‐HT) and macronutrient selection have been more fruitful (Fadda, 2000; Weingarten & Elston, 1991).

Nutritional research by Gessa, Biggio, Fadda, Corsini, and Tagliamonte (1974) led to the conclusion that the brain's general function is influenced by nutrients in each individual's diet. Studies on rats demonstrated that a blend of essential amino acids which was tryptophan‐free (TRP) caused significant reductions in the brain's TRP as well as 5‐HT levels (Gessa et al., 1974). Large neutral amino acids (LNAAs) compete for the same transport system as TRP; thus, larger concentrations of one amino type (e.g., phenylalanine) imply smaller quantities of the other (Biggio, Fadda, Fanni, Tagliamonte, & Gessa, 1974; Gessa, Biggio, Fadda, Corsini, & Tagliamonte, 1975; Pardridge, 1986; Wurtman, Hefti, & Melamed, 1981; Young et al., 1988). Thus, if the main building block for 5‐HT synthesis is removed, levels of serotonin drop significantly, lower than the norm (Fadda, 2000). Therefore, using nonpharmacological manipulations, it is possible to study how 5‐HT deficits affect nutritional preferences in human and nonhuman subjects.

5‐HT concentration in the brain is particularly affected by three dietary elements: fatty acids, proteins, and carbohydrates. Albumin proteins in the plasma act as a transport system for various molecules including TRP. However, amino acids that bind to albumin cannot pass the blood brain barrier (BBB). The solution for this is the intake of fatty acids which have a strong binding attraction for albumin proteins, eventually leaving more TRP to cross the BBB and get converted to 5‐HT (Sainio, Pulkki, & Young, 1996). Another factor of TRP availability in the plasma is also protein consumption. Studies on rodents argue that TRP and 5‐HT levels decrease due to other competing LNAAs for the BBB. On the other hand, carbohydrate consumption leads to the opposite effect. Insulin increase in the blood stimulates the uptake of LNAAs but not TRP. This decreases the competitors of TRP for the BBB as well as the plasmatic concentration of other LNAAs leading to more 5‐HT production (Maher, Glaeser, & Wurtman, 1984; Pardridge & Oldendorf, 1975).

In support of the above, Fadda (2000) postulated that laboratory animals treated with a TRP‐free amino acid mixture had a preference for carbohydrates, whereas when they had a glucose meal supplement with TRP, a preference for proteins was observed. Furthermore, Morris, Li, MacMillan, and Anderson (1987) observed that rats treated with TRP had a significant decrease in overall food intake, with carbohydrate decrease being slightly significantly greater than proteins. Moreover Young et al. (1988) exposed two groups of individuals to a large buffet after orally administering a TRP‐free and a TRP‐balanced mixture; participants who had the TRP‐free mixture had a small but significant decline in protein selection with no difference in the preference of fats, carbohydrates, or total calories. These results show that in humans, 5‐HT is implicated in the control of protein selection. Anorexia has been characterized by increased extracellular 5‐HT due to decreased levels of its metabolite 5‐hydroxyindoleacetic acid. Intake of carbohydrates or fatty foods (highly aversive for anorexics), leads to increased uptake of TRP in the brain increasing extracellular 5‐HT associated in return with heightened anxiety and dysphoria. Thus, starvation in this disorder might represent a way of lowering 5‐HT levels resulting in a short‐lived emotional balance (Kaye, Fudge, & Paulus, 2009).

The evidence to date suggests that tryptophan, the precursor of serotonin, has a role to play when it comes to food or macronutrient selection. Ceci et al. (1989) examined 5‐HT in relation to eating behavior, weight loss as well as diet compliance. They observed that obese females, when administered with 5‐HTP (8 mg kg^−1^ day^−1^) for a period of 35 days, not only lose weight and decrease their food intake but they also show anorexia‐related symptoms (e.g., early satiety). Furthermore, normalizing depressed brain TRP levels with additional supplementation can lead to weight loss in obese patients (Cangiano et al., 1992, 1998).

Consideration of the brain's reactions to food stimuli demonstrates that responses are not restricted to a single area but rather a complex network of structures that participate in the evaluation of calories, nutrients, hunger, or satiety (Tataranni et al., 1999). Functional magnetic resonance imaging (fMRI) and positron emission tomography (PET) illustrate that responses to food cues depend highly on an individual's physiological brain states. Factors such as hunger, satiety, and the palatability of high‐energy foods—all affect food preferences (Laan, Ridder, Viergever, & Smeets, 2011; LaBar et al., 2001; Pelchat et al., 2004). One of the central orexigenic regions of the brain that was shown to be responsible not only for the initiation, but also for the termination of meals is the hypothalamus (King, 2006). The hypothalamus projects to the somatosensory cortex and the insula which is responsible for intra‐sensory and extrasensory taste perception (Doyle, Rohner‐Jean, and Jeanrenaud, 1993; Rolls, 2000).

Cortical regions responsive to food images include the limbic and paralimbic areas (Tataranni et al., 1999). Part of this system is the prefrontal cortex (PFC) which by receiving inputs from the hypothalamus, is implicated in food‐seeking behaviors as well as acting as a “regulating valve” for food consumption. Neuroimaging studies argue that the ventromedial PFC (VMPFC) and dorsolateral PFC (DLPFC) in particular, exhibit an increased BOLD response during satiation in comparison to hunger (Del Parigi et al., 2002; Simmons, Martin, & Barsalou, 2005; Tataranni et al., 1999).

The insula, characterized by neurological studies as the site of the primary taste cortex, is of considerable interest since it connects the hypothalamus, OFC, the limbic system, and the occipital cortex (Cerf‐Ducastel, Van de Moortele, MacLeod, Le Bihan, & Faurion, 2001; Schienle et al., 2002; Tataranni et al., 1999). Individuals treated with monotonous diets when presented with non‐diet food stimuli demonstrated more activity in the left insula signifying craving and food desirability‐related activation (Pelchat et al., 2004). Tataranni et al. (1999) have also shown the insular cortex to be associated with conditions of hunger. Also within the limbic system, the amygdala and cingulate cortex not only show a positive correlation with hunger but they also display sensitivity to food desirability (Arana et al., 2003; Shin, Zheng, & Berthoud, 2009). For instance the anterior cingulate shows an inverse relation with the desirability of chocolate, higher functional activity for high versus low caloric food, as well as greater activity in satiation compared to hunger (Killgore & Yurgelun‐Todd, 2005; Santel, Baving, Krauel, Munte, & Rotte, 2006; Small, Zatorre, Dagher, Evans, & Jones‐Gotman, 2001).

The hippocampus and the parahippocampal gyrus BOLD show enhanced responses in craving‐related situations and in states of hunger (Davids et al., 2009; Tataranni et al., 1999). These regions being structures of the memory system have a major role in the identification and assimilation of internal energy states regulating the feeding behavior of individuals through effectively evaluating stimuli (Berthoud, 2002; Davids et al., 2009; Davidson & Jarrard, 1993).

Within the basal ganglia, the caudate and putamen are both regions involved in the evaluation of reward and they show opposing activation in states of hunger and satiety (Holsen et al., 2005; Pelchat et al., 2004). The putamen, in particular, is implicated in Pavlovian conditioning (O'Doherty et al., 2004) and it has been postulated by Davids et al. (2009) that activity of these regions below the norm might represent a difficulty in correct activation of the reward pathway in obese children. The dorsal striatum, in a meta‐analyses by van Meer, van der Laan, Adan, Viergenever, and Smeets (2014), has been suggested to maintain caloric requirements for survival as well as food motivation.

Food‐related responses have also been identified more broadly within the parietal and temporal lobes. Within the area of eating disorders, the inferior parietal lobe (IPL) has shown differential activity between anorexic individuals and controls (Santel et al., 2006). This impaired activity of the IPL might represent the fasting state of anorexics as well as a decreased “somatosensory‐gustatory responsiveness” inversely related with the over‐activity of this region observed in obese individuals (Karhunen, Lappalainen, Vanninen, Kuikka, & Uusitupa, 1997; Wang et al., 2002). Furthermore, the postcentral gyrus of the IPL (BA 1, 2, 3) contains body representation parts such as the tongue, teeth, and lips (Deuchert et al., 2002; Heimer, 1995). The most reactive region of the temporal lobe in hungry versus non‐hungry states is the fusiform gyrus (FG; Fisher, 1994; LaBar et al., 2001; Santel et al., 2006). The FG has been reported to be more responsive in low compared to high calorie food (Killgore & Yurgelun‐Todd, 2005). In addition Davids et al. (2009) argued that normal individuals compared with obese, showed more activity in the FG when viewing food. Finally, the middle temporal gyrus usually involved in the perception of emotional stimuli has also shown to be responsive to food stimuli in states of satiety (Mourao‐Miranda et al., 2003; Santel et al., 2006).

This study was designed to examine which regions of the brain are functionally active in response to food‐relevant content when individuals are treated with 5‐HTP versus a vitamin C control, and while in a state of hunger. Furthermore cortical activity between the two groups will be examined (Control vs. 5‐HTP) as well as cortical responses based on the caloric and nutrient content of food (high calories/carbohydrates vs. proteins and proteins vs. high calories/carbohydrates). Based on previous literature, the following hypotheses are proposed:

When both groups are combined, the central orexigenic network—the left inferior frontal gyrus, the basal ganglia, the parietal and temporal regions as well as LOC—will be activated in response to food visual stimuli.
Since 5‐HTP is associated with decreased appetite, early satiety anorexia‐related symptoms as well as weight loss, it is reasonable to expect that relevant regions will show more activity in the 5‐HTP group compared to controls. Thus, it is hypothesized that compared to controls, the 5‐HTP group activity will show pronounced activity in the frontal cortices, the cingulate cortex, the caudate, putamen, FG, and hippocampal/parahippocampal structures. In addition, the temporal cortex is likely to show activity since it has been linked with the satiation domain. Moreover, the left lingual gyrus (LG) is expected to show increased activity, whereas the right LG decreased activity since this region has been associated with anorexia‐related symptoms and appetite inhibition.
(i) The cortical activity of the 5‐HTP group compared to controls will be greater for protein stimuli than carbohydrates and high calories. Thus, it is expected that the PFC of individuals in the 5‐HTP group compared to controls will be more active when viewing carbohydrates and high calories compared to proteins indicating suppression of unwanted engagement. (ii) In contrast, the control group (in comparison to the 5‐HTP group) should show a pronounced PF activity in the proteins condition since this food category is not as desirable as the high calories/carbohydrates conditions.
(i) Protein stimuli contrasted with carbohydrates/high calories in the 5‐HTP versus control group are expected to show no prefrontal activity. The amygdala should show increased BOLD responses due to this region's sensitivity to food desirability. Furthermore, the parietal lobe (BA 40) is likely to show more activity illustrating imaginary taste and texture perception of food desirability. Also due to food desirability, the FG should show more activity because increased activity of this region has been associated with heightened attention and increased visual processing. (ii) Similar functional activity is expected for the control versus 5‐HTP group but for the reversed contrast (high calories and carbohydrates > proteins).

## 2 Methods

### 2.1 Participants

All six males and eight females who participated were from a mixed cultural background, did not suffer from any physical, neurological, mental, or eating disorder and were free of any medication. All participants were in their mid 20s. Prior to MRI imaging, local ethical approval was obtained and each participant was screened in accordance with standard procedures, and informed consent was given.

### 2.2 Design, stimuli, and materials

**Figure 1:**
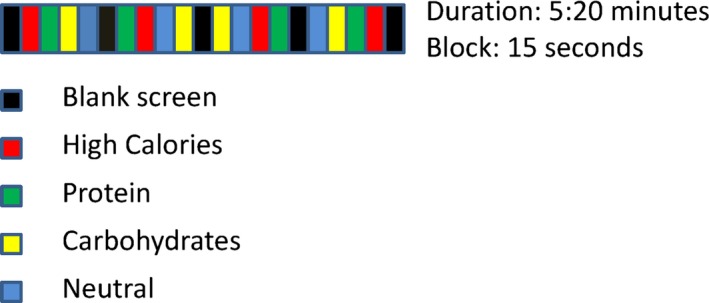
Illustration of the block design timecourse, including the sequence and time intervals of stimulus presentation

A block design approach was utilized, with stimulus blocks presented for 15 s each, and a total simulation time of 5 min 20 s. Four stimulus categories were presented using Microsoft Power Point in a counterbalanced order along with a blank screen that acted as a reference point (Figure 1). These comprised protein, high‐calorie, and carbohydrate stimuli as well as nonfood objects as a control. Video excerpts were selected as stimuli rather than desirable food names or pictures since short clips are closer to real experiences (Davids et al., 2009; LaBar et al., 2001; Pelchat et al., 2004). The stimuli were taken from YouTube, of which 70% consisted of TV commercials and the rest were taken from cooking shows. Each one was cropped and resized prior to the experiment. Protein stimuli were made up of different types of meat (beef, chicken, lamb, and pork) cooked on a grill. The high calories category included a combination of fatty and carbohydrate‐rich foods such as fast food, whereas the carbohydrates category included plain pasta, bread, and whole grain cereals. The neutral nonfood stimuli included elements such as landscapes, cars, and helicopters.

In order to manipulate the 5‐HTP levels of each individual, commercially available capsules were used (manufacturer: Doctor's Best) which consisted of modified cellulose (veggie cap), rice powder, and magnesium stearate (vegetable source). The administered dose (100 mg) did not exceed the daily recommended dose which is approximately 4 mg/kg body weight (e.g., 280 mg for an adult weighting 70 kg; Elango & Pencharz, 2009). A vitamin C capsule (125 mg) was used for the control condition. Vitamin C was thought to be a reasonable placebo particularly for this study due to the fact that it has not been suggested to mediate food selection (Cangiano et al., 1992, 1998; Morris et al., 1987). Conventional placebo pills that have been used in other pharmacological research were sugar pills (dextrose), over the counter analgesics as well as vitamins (Tilburt, Emanuel, Kaptchuk, Curlin, & Miller, 2008). Since caloric intake was the main focus of this study, the placebo used did not have any active ingredients that could have contaminated the data, or intervened with the transmission of tryptophan to the brain such as fatty acids (insulin spikes Golomb et al., 2010), or contain traces of tryptophan (e.g., rice, oats) (Gerhardt & Gallo, 1998). Thus, a placebo (vitamin C) was chosen that had no reported side effects and had no impact on food consumption and caloric intake. Both groups received their experimental condition dissolved in 13 cl of orange juice to ease swallowing (Mohammad & Siddiqui, 2011).

### 2.3 Procedure

All individuals were separated randomly into two groups of which they were completely unaware. Half of them received the food supplement, 5‐HTP, and the rest received a vitamin C tablet dissolved in orange juice. The day before the study, participants were asked to fast overnight (excluding dietary supplements/medication) and abstain from alcohol for at least 24 hr. The supplements were taken on an empty stomach 30–45 min prior to scanning in order to ensure digestion (Cangiano et al., 1992, 1998). Prior to scanning, participants were asked to imagine their favorite food for 2 min and focus on texture, smell, and taste (Pelchat et al., 2004). Their responses were collected and a particular focus was given on the description of the food. The caloric content of the recalled food was averaged from the following sources according to average portions: (1) Cheyette and Ballolia (2010), Carbs & Cals & Protein & Fat. A visual guide to Carbohydrate, Protein, Fat & Calorie Counting for Healthy Eating & Weight Loss; (2) McCance and Widdowson's (2004), The Composition of Foods, 6th ed. Food Standards Agency; and (3) Nutritics (2011), Nutritics professional diet analysis software (Available from: http://www.nutritics.com/). During the fMRI scanning session, participants were instructed to passively observe the stimuli presented on the screen.

### 2.4 fMRI recording parameters

All images were acquired on a 3T Siemens Magnetom Trio scanner using a standard eight‐channel array head coil. Functional images were acquired in an axial orientation with a standard gradient echo, echoplanar sequence (39 slices, voxel size 3 × 3 × 3 mm, TR of 3,000 ms, TE 33 ms, 64 × 64 matrix, flip angle of 90°). A high resolution, three dimensional T1 anatomical image (1 × 1 × 1 mm) of the whole brain was also acquired for visualization (TR 1,900 ms, TE 5.57 ms, flip angle 11°).

### 2.5 Data analysis

Analysis of all fMRI data was carried out using SPM8 (RRID:SCR_007037 http://www.fil.ion.ucl.ac.uk/spm). Functional images were realigned and coregistered with the structural image, correcting for any involuntary movements that might have occurred during the scanning session. In addition, both functional and structural images were normalized to the Montreal Neurological Institute (MNI) standard space. Prior to statistical analysis, functional images were smoothed using a 6‐mm FWHM Gaussian kernel. For the block design, the four stimulus conditions (high calories, proteins, carbohydrates, neutral) were modeled as separate regressors within a general linear model. Prior to a random‐effects group analysis, subject‐specific parameter estimates for each regressor were calculated along with appropriate condition‐specific contrasts. Subsequently, the following second‐level comparisons were made which are reported at *p* < .001 (uncorrected) due to the limited number of participants (cluster activation surviving FWE correction at *p* < .05 are also indicated where possible):

1. In order to determine which regions of the brain show a preferential response to food compared to random (neutral) visual stimuli, one‐sample *t* tests were conducted for each group (vitamin C and 5‐HTP) comparing ([protein + carbohydrates + high calories] vs. [neutral]).
2. A two‐sample *t* test was conducted in order to examine whether there was a different BOLD response between the groups (vitamin C vs. 5‐HTP) in the observation of food (i.e., protein + carbohydrates + high calories) compared to neutral stimuli.
3. Finally, in order to observe whether proteins (proteins > carbohydrates and high calories) or carbohydrates and high calories (carbohydrates and high calories > proteins) show regionally different activity for the 5‐HTP and vitamin C group, four separate one‐sample *t* tests were conducted.

For the reporting of results, MNI coordinates were used and the coordinates for each region were presented in the following manner—RH (right hemisphere)/LH (left hemisphere) (coordinates: *x*,* y*,* z*).

### 2.6 Ethical standards

All procedures followed were in accordance with the ethical standards of the responsible committee on human experimentation (institutional and national) and with the Helsinki Declaration of 1975, as revised in 2000 (5). Informed consent was obtained from all patients for being included in the study.

## 3 Results

### 3.1 The orexigenic network for vitamin C and 5‐HTP group

**Figure 2:**
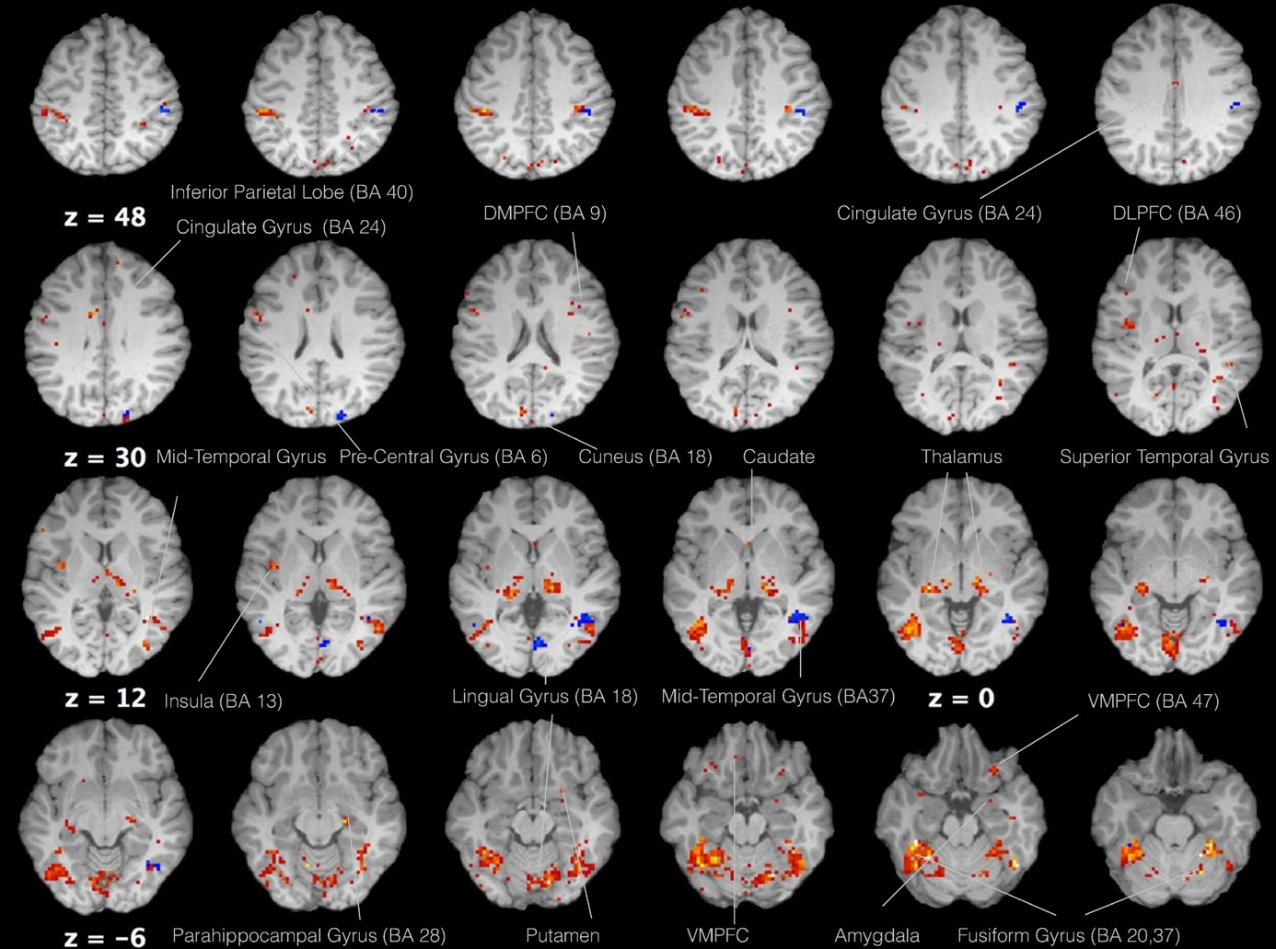
Axial view of regions that showed food selective activity ([protein + carbohydrates + high calories] vs. neutral) for both 5‐HTP (red) and vitamin C (blue) groups (voxel threshold *T *=* *3.85, *p *<* *.001)

A one‐sample *t* test was conducted for both the 5‐HTP and control group to examine differences in response to food versus neutral visual stimuli. The 5‐HTP group showed bilateral activity at the posterior FG (BA 20, 37) with maximum activation in the left hemisphere. Activation clusters were located at the inferior right LG (BA 18) that stretched dorsally and contralaterally to the left LG. Moreover activation clusters were observed in the parietal lobe, particularly, the right primary motor cortex (BA 4), bilaterally at the lateral premotor cortex (BA 6) as well as the supramarginal gyrus (BA 40). Limbic structures that demonstrated bilateral activation included the thalamus, the insula (BA 13), the VMPFC (BA 47), and the cingulate gyrus (BA 24). Activated limbic structures that were restricted to one hemisphere were the right parahippocampal gyrus (BA 28) and the amygdala. Furthermore, frontal lobe activity was observed on the dorsomedial frontal gyrus (BA 9) bilaterally and the left DLPFC (BA 46). Bilateral basal ganglia activity was observed in the putamen and the right caudate. Finally, multiple regions of the occipital and temporal lobe showed positive responses to food stimuli such as the mid temporal gyrus, the superior temporal gyrus bilaterally, the left middle occipital gyrus (BA 19), the precuneus, and the cuneus bilaterally (Table 1, Figure 2).

**Table 1:** Regions active in response to food after 5‐HTP consumption

Region	Brodmann's area	MNI atlas coordinates			*Z* score
		*x*	*y*	*z*	
Posterior fusiform gyrus*	20, 37	−39	−40	−17	4.71
		24	−52	−20	4.46
Lingual gyrus*	18	15	−76	−11	4.69
		−3	−82	43	3.47
Thalamus*		−27	−28	1	4.42
		18	−28	7	4.29
Middle temporal gyrus*		39	−55	13	4.38
	21	45	−19	−17	3.52
Inferior parietal lobe*	40	−42	−31	43	4.30
		39	−28	40	3.92
Parahippocampal gyrus	28	24	−22	−8	4.19
Cingulate gyrus	24	−9	2	31	4.00
		12	−46	25	3.49
Insula*	13	−39	−10	10	3.83
		51	−16	25	3.76
Cuneus	18, 19	−9	−85	25	3.80
		21	−91	34	3.31
Precentral gyrus	4, 6	−57	2	28	3.69
		36	2	25	3.36
		33	−31	58	3.33
Precuneus		9	−76	43	3.66
		−24	−73	43	3.58
Superior Parietal lobe		30	−52	70	3.61
VMPFC	47	30	23	−17	3.57
		−30	29	−14	3.21
		−9	35	−14	3.39
Superior temporal gyrus		48	−43	16	3.55
Caudate		3	11	4	3.53
Cingulate gyrus	24	0	−7	34	3.38
Superior temporal gyrus		−33	2	−17	3.34
Amygdala		27	−4	−17	3.34
Putamen		27	5	−11	3.32
		−18	14	−5	3.22
DLPFC	9, 46	42	9	25	3.21
		−39	23	22	3.21
		−42	20	16	3.17

Table shows peak voxel coordinates for activation clusters at *p* < .001 uncorrected. Cluster activation surviving FWE correction at *p* < .005 are indicated (*).

For the vitamin C group activity was rather more limited, located in regions of the parietal, temporal, and occipital lobes. More specifically, these included the right superior (BA 7) and IPL (BA 40), the middle temporal gyrus bilaterally (BA 37), the LG bilaterally (BA 18), and the right superior occipital lobe (Table 2, Figure 2).

**Table 2:** Regions active in response to food for the vitamin C condition

Region	Brodmann's area	MNI atlas coordinates			*Z* score
		*x*	*y*	*z*	
Superior parietal lobe	7	33	−52	70	3.94
Lingual gyrus	18	6	−76	7	3.8
Middle temporal gyrus*	37, 39	42	−55	1	3.79
		−51	−58	10	3.76
Inferior parietal lobe	40	51	−31	49	3.6
Fusiform gyrus	20	−39	−43	−20	3.18

Table shows peak voxel coordinates for activation clusters at *p* < .001 uncorrected. Cluster activation surviving FWE correction at *p* < .05 are indicated (*).

### 3.2 Cortical influences of 5‐HTP on food stimuli

**Figure 3:**
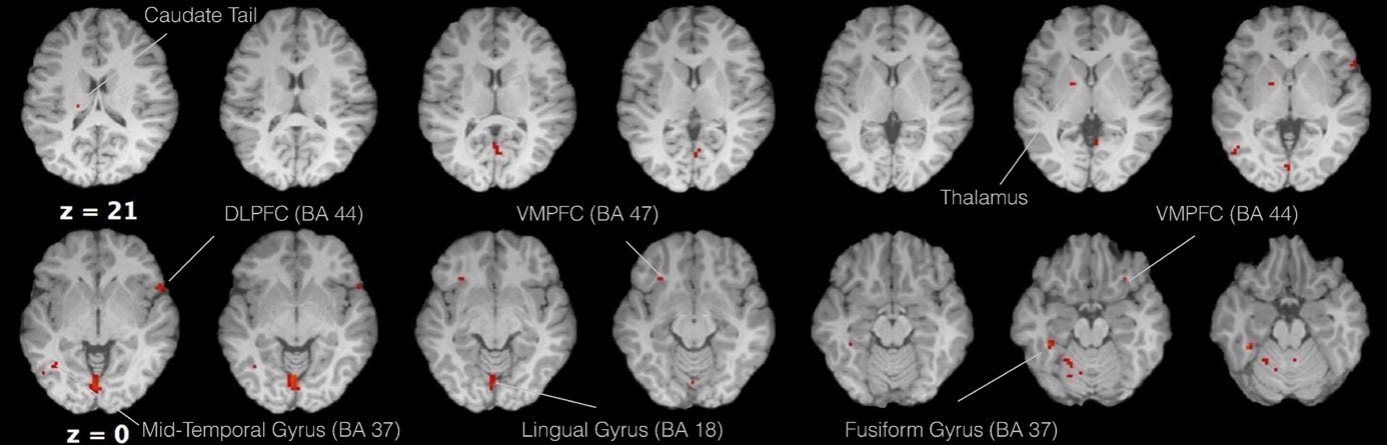
Axial view of regions that showed greater activity in the 5‐HTP versus vitamin C comparison, in the food versus neutral condition *(*voxel threshold *T *=* *3.92, *p *<* *.001)

A two‐sample *t* test between the 5‐HTP and control group, examining differences in responses to the food versus neutral contrast showed significantly greater active regions for the 5‐HTP group. Maximum activity was observed in the LG (BA 18) neighboring with the posterior cingulate. Activation clusters were also observed in the FG (BA 37), which extended ventrolaterally and caudally into the cerebellar culmen and anteriorly in the parahippocampal gyrus (BA 36). Limbic structures such as the caudate tail, the thalamus as well as regions of the frontal lobe showed a functional response. On the frontal lobe, active voxels were observed on the VMPFC (BA, 44, 47) bilaterally, the right DLPFC (BA 44), the paracentral lobule, the medial frontal gyrus (BA 6), the middle frontal gyrus, and finally, the superior frontal gyrus (BA 6). Activation was also observed in regions of the parietal lobe such as the precuneus (BA 7) as well as the middle temporal gyrus (BA 37) (Table 3, Figure 3). The reverse test for differences between the control and 5‐HTP group did not show any significant differences at the defined threshold of *p* < .001 (uncorrected).

**Table 3:** Regions that showed greater activity in the 5‐HTP condition compared to controls in response to food

Region	Brodmann's area	MNI atlas coordinates			*Z* score
		*x*	*y*	*z*	
Lingual gyrus	18	−3	−73	−2	4.21
		6	−61	7	3.13
Fusiform gyrus	37	−39	−40	−14	4.08
Caudate tail		−18	−25	22	3.69
Thalamus		−15	−4	7	3.66
DLPFC	44	60	14	1	3.59
VMPFC	44, 47	33	23	−14	3.51
		−33	23	−8	3.45
Precuneus	7	−12	−76	52	3.55
		21	−73	43	3.43
Mid temporal gyrus	37	−39	−61	1	3.43
Medial frontal/paracentral gyrus	6	9	−25	67	3.31
		6	−22	73	3.21
		9	11	49	3.20
Superior frontal gyrus	8	45	26	46	3.11

Table shows peak voxel coordinates for activation clusters at *p* < .001 uncorrected.

### 3.3 Regional activity according to food category

**Figure 4:**
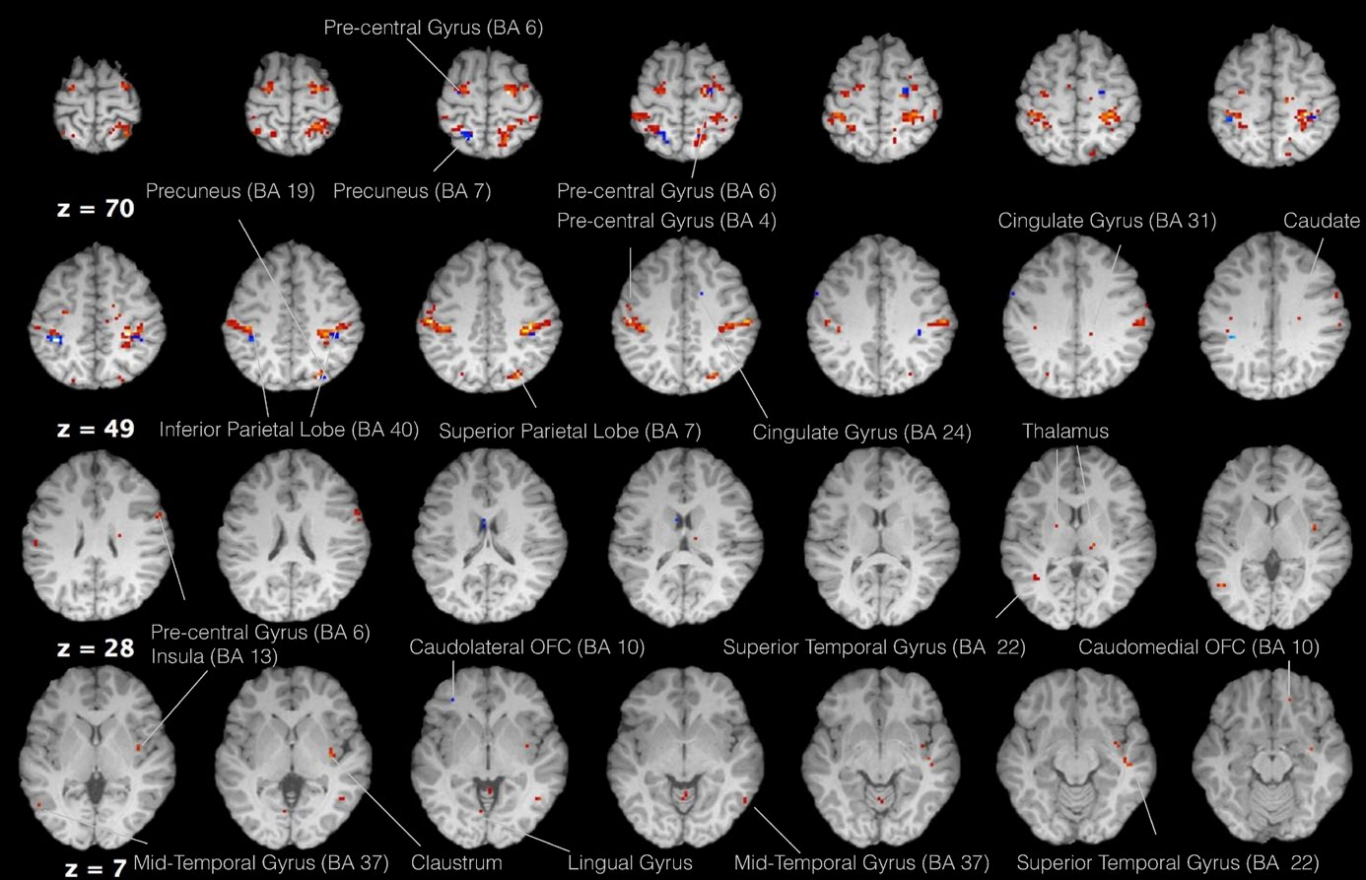
Axial view of regions that showed activity in the protein > high calories and carbohydrates contrast for the vitamin C (blue) and 5‐HTP (red) groups (voxel threshold *T *=* *5.2, *p *<* *.001)

In order to observe how food content and 5‐HTP consumption affect cortical function, category‐specific one‐sample *t* tests ([protein > high calories and carbohydrates] and [high calories and carbohydrates > protein]) were performed for both the 5‐HTP and control group. In the case of the 5‐HTP group, the area of maximum activation in favor of protein‐rich food was the right precentral gyrus (BA 6). The same activation pattern was also observed on the left precentral gyrus (BA 6), which started dorsally and extended ventrolaterally with some isolated clusters situated rostrally in BA 4. An extensive cluster area was situated bilaterally at the superior parietal lobe (BA 7), which extended laterally and rostroventrally into the IPL (BA 40). In addition, limbic structures that showed activity in favor of food rich in protein content were the thalamus bilaterally, the insula, the right cingulate gyrus, and the right caudomedial OFC (BA 10). Moreover, parts of the basal ganglia that were observed to be active included the right claustrum and the right caudate. Finally, activity was observed on both temporal and occipital lobes such as the superior temporal gyrus (BA 22) bilaterally, the right (BA 21) and left middle temporal gyrus (BA 37) as well as the left LG and left precuneus (BA 7). In the case of the vitamin C group, activity was in general less pronounced in relation to protein‐rich food. The area of maximum activation was the left IPL (BA 40). This area was also evident on the right hemisphere but to a lesser extent. Furthermore, bilateral activity was observed on the precentral gyrus (BA 6), the left (BA 34) and right cingulate gyrus (BA 24), the left superior parietal lobe (BA 7), the right precuneus (BA 19) as well as the left ventrolateral OFC (BA 10). (Tables 4 and 5, Figure 4).

**Table 4:** Regions active in response to food high in proteins for the 5‐HTP group

Region	Brodmann's Area	MNI atlas coordinates			*Z* score
		*x*	*y*	*z*	
Precentral gyrus*	4, 6	21	−13	64	5.25
		−21	−10	67	3.84
		−51	−10	40	3.41
Inferior parietal lobe*	40	42	−28	43	4.81
		−54	−22	43	4.73
Superior parietal lobe*	7	27	−76	46	4.73
		−30	−46	55	3.78
Middle temporal gyrus	37	−48	−64	10	4.03
		60	−58	−2	3.23
Insula/claustrum	13	39	−16	4	3.93
		39	−10	11	3.65
Superior temporal gyrus	22	51	−25	−8	3.68
		−39	−55	13	3.22
Thalamus		18	−25	13	3.64
		−18	−7	13	3.29
Caudomedial orbital frontal cortex	10	18	38	−11	3.49
Caudate		27	−19	31	3.42
Lingual gyrus		−6	−70	1	3.13
Cingulate gyrus	31	15	−34	34	3.11

Table shows peak voxel coordinates for activation clusters at *p* < .001 uncorrected. Cluster activation surviving FWE correction at *p* < .05 are indicated (*).

**Table 5:** Regions active in response to food high in proteins for the vitamin C group

Region	Brodmann's area	MNI atlas coordinates			*Z* score
		*x*	*y*	*z*	
Inferior parietal lobe	40	−39	−40	49	4.63
		36	−37	52	3.34
Precentral gyrus	6	24	−10	58	3.5
		−27	−13	64	3.2
Caudolateral orbital frontal cortex	10	−33	38	1	3.2
Precuneus	7, 19	−18	−55	64	3.5
		33	−76	46	3.2
Cingulate gyrus	24, 32	18	5	40	3.16
		−15	14	34	3.09

Table shows peak voxel coordinates for activation clusters at *p* < .001 uncorrected.

**Figure 5:**
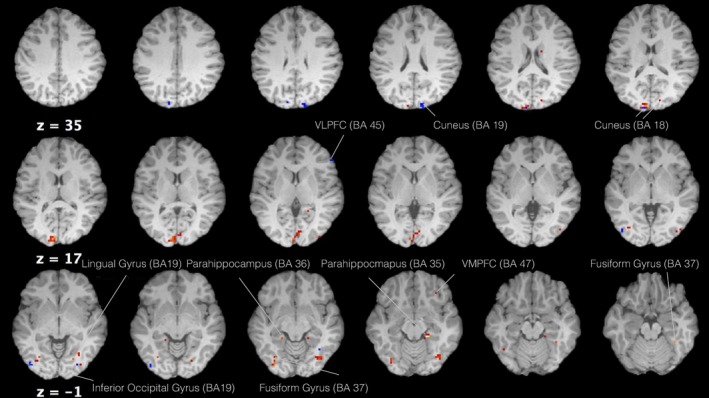
Axial view of regions that showed activity in the high calories and carbohydrates > protein contrast for the vitamin C (blue) and 5‐HTP (red) groups (voxel threshold *T *=* *5.2, *p *<* *.001)

Examining the opposite contrast for both groups, cortical activity was generally less extensive in response to food high in caloric value and carbohydrates. The right parahippocampal gyrus (BA 35) was the region of maximum activation for the 5‐HTP group, and was also present at the left hemisphere (BA 36). At the frontal lobe, activity was located at the right VMPFC (BA 47) and at the superior frontal gyrus (BA 8). Moreover, activation clusters were present bilaterally at the inferior (BA 19) and middle occipital gyrus (BA 18) as well as the posterior inferior temporal gyrus (BA 37). In the case of the control group, the area of maximum activation was the right cuneus (BA 19). Bilateral activity was present at the inferior (BA 19) and middle occipital gyrus (BA 18), the posterior inferior temporal gyrus (BA 37) as well as the VLPFC (Tables 6 and 7, Figure 5).

**Table 6:** Regions active in response to food high in carbohydrates and calories for the 5‐HTP group

Region	Brodmann's area	MNI atlas coordinates			*Z* score
		*x*	*y*	*y*	
Parahippocampus	35	21	−37	−8	4.78
		−21	−40	−5	3.61
Cuneus*	18	−6	−94	22	4.02
		15	−85	22	3.24
Fusiform gyrus	37	39	−46	−14	3.91
		−30	−70	−5	3.59
Lingual gyrus	19	24	−73	1	3.72
Superior Frontal Gyrus	6	−18	32	58	3.50
VLPFC	47	36	32	−8	3.22

Table shows peak voxel coordinates for activation clusters at *p* < .001 uncorrected. Cluster activation surviving FWE correction at *p* < .05 are indicated (*).

**Table 7:** Regions active in response to food high in carbohydrates and calories for the vitamin C group

Region	Brodmann's area	MNI atlas coordinates			*Z* score
		*x*	*y*	*x*	
Inferior occipital gyrus/cuneus	18, 19	15	−94	28	3.53
		−42	−82	1	3.52
		−12	−97	22	3.39
		30	−82	1	3.2
Fusiform gyrus	37	39	−58	−5	3.27
VLPFC	45	60	32	13	3.19

Table shows peak voxel coordinates for activation clusters at *p* < .001 uncorrected.

### 3.4 Cephalic phase caloric group comparison

**Figure 6:**
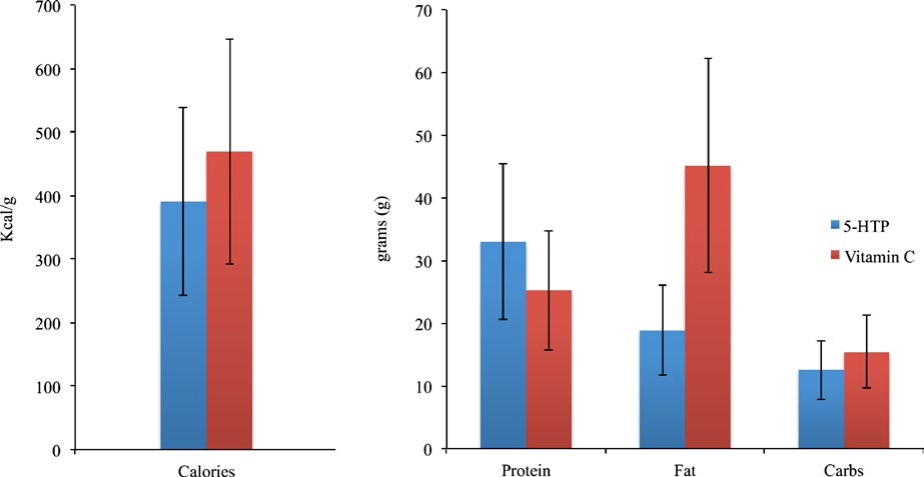
Bar chart representing the mean caloric difference of the two groups as well as the grams of proteins, carbohydrates, and fats in each food recalled prior to scanning

Responses were collected prior to the scanning session (cephalic phase) in order to observe whether there was a significant difference between the 5‐HTP and control group in total calories as well as the nutritional content (protein, fat, carbohydrate) of food recalled (Table 8, Figure 6). An independent samples *t* test showed no significant difference in the caloric mean score between controls and the 5‐HTP group. No significant difference was observed in the grams of fats, carbohydrates, and proteins between the two groups.

**Table 8:** Individuals’ food responses during the cephalic phase representing portions, calories, protein , carbohydrate, and fat content

Condition	Cephalic phase	Calories	Protein (g)	Carbs (g)	Fat (g)	Average gr.
5‐HTP	Steak	315	37	0	11	110
5‐HTP	Steak	315	37	0	11	110
5‐HTP	Steak	315	37	0	11	110
5‐HTP	Curry with prawns, rice, butternut squash	350	16	50	15	350
5‐HTP	2 × eggs benedict	848	54	64	14	2 (pieces)
5‐HTP	Japanese curry with vegetables	280	13	18	15	225
5‐HTP	Steak	315	37	0	11	110
Vitamin C	Pepper steak with mash potatoes	431	29	14	0	325
Vitamin C	Lahmatzu	576	20	64	12	300
Vitamin C	Pasta	230	8	49	2	220
Vitamin C	Steak, chips, and fried eggs	912	60	59	49	395
Vitamin C	Rack of ribs	375	26	18	22	250
Vitamin C	Pizza (average)	535	26	63	21	217
Vitamin C	Pasta	230	8	49	2	220

## 4 Discussion

The food‐selective cortical network was activated in response to the combined stimulation of high calorie, carbohydrate, and protein stimuli versus neutral stimuli. In agreement with findings by Fuhrer, Zysset, and Stumvoll (2008) as well as Tataranni et al. (1999), bilateral activity was observed in the thalamus, a diencephalic region considered as the cornerstone of an eating episode since it links primary taste and sensorimotor cortices with frontal regions of behavioral engagement (Doyle, Rohner‐Jean, and Jeanrenaud, 1993; Rolls, 2000; Shin et al., 2009). Receiving projections from the thalamus, hemodynamic responses were observed in regions of emotional processing: The left middle insula has been suggested to be an area representing the imaginary perception of texture, smell, and taste (De Araujo & Rolls, 2004; Pelchat et al., 2004) as well as being responsible for the memory retrieval of previous food experiences (Pelchat et al., 2004). The role of the middle insula (BA 13) in the orexigenic network is not only crucial for sensory evaluation but also for the recall of food experiences and enteroceptive monitoring (Killgore et al., 2003; Pelchat et al., 2004; Tataranni et al., 1999). Closely linked with the above structure are secondary somatosensory areas (BA 40) that together complete the network of extrasensory taste perception and gustatory responsiveness (Doyle, Rohner‐Jean, and Jeanrenaud, 1993; Fuhrer et al., 2008; Laan et al., 2011; Pelchat et al., 2004; Rolls, 2000). In addition, the superior parietal gyrus (BA 7), an area involved in multisensory integration has been suggested to act along with the left inferior frontal gyrus (BA 45) as a subjective rating measure of mentally perceived sensations of primary gustatory and taste cortices. Siep et al. (2009) have argued that explicit processing of food is carried out by the amygdala and the medial orbital gyrus as increased activation of these regions are observed when participants are asked to judge the palatability of food. In general, the amygdala seems to have extended roles in the orexigenic network, as in conjunction with the lateral orbital gyrus (BA 47) it has been proposed to modulate states of hunger (Laan et al., 2011), whereas when taken together with the cingulate gyrus, it has been suggested to be involved in food desirability and the motivational salience of food (Arana et al., 2003; Shin et al., 2009). This is probably because of its strategic position in the cortex. The parahippocampal gyrus on the other hand is not only part of reward processing but also reward integration (Pelchat, et al., 2004; Small et al., 2001; Tataranni, et al., 1999). The middle temporal gyrus (Santel et al., 2006) and the parahippocampal gyrus (Breiter et al., 1997; Tataranni et al., 1999) are thought to play a crucial role in reward evaluation and feeding behavior as they store information about the energy states of external sensory cues, integrating them with internal organismic needs. Moreover, the extensive activation observed in the lateral occipital cortex (Cuneus and FG) is believed to be associated with the arousing value of food (Santel et al., 2006). These regions implicated in object recognition show increased activity as the derivative of heightened attention and increased emotional processing (Killgore & Yurgelun‐Todd, 2007). Finally, it is important to note that subcortical structures such as the parahippocampal gyrus, thalamus, cingulate gyrus, insula, caudate, and putamen, feed caloric and sensory information of external stimuli to the IFG (BA 45, 47) and the middle frontal gyrus (BA 9, 10, 46) which plan behavioral goal engagement or control according to internal physiological needs (Fuhrer et al., 2008; Laan et al., 2011; Tataranni et al., 1999). Overall the activity observed in the 5‐HTP group in response to food stimuli compared to the vitamin C group was more pronounced. Activity of the vitamin C group in response to the general food category was restricted in regions of the temporal, parietal, and occipital lobe responsible for memory recall, specialized object recognition, and sensory perception. No activity was observed in limbic regions or frontal cortices responsible for planning, evaluation, enteroceptive awareness, and goal engagement. Nevertheless, for the ([protein > high calories and carbohydrates] and [high calories and carbohydrates > protein]) contrasts scarce limbic and frontal lobe activity was observed for the vitamin C group.

The cerebral influence of 5‐HTP intake to food stimuli seems to share similarities with studies that examined brain activity in altered states of hunger (Santel et al., 2006; Small et al., 2001) and in individuals with a clinical weight profile (Cangiano et al., 1992, 1998; Ceci et al., 1989; Cornier et al., 2009; Davids et al., 2009; Santel et al., 2006). The results obtained in the 5‐HTP group are consistent with observations made by Davids et al. (2009) who compared obese and lean children. Obese children had difficulties activating regions related to reward evaluation and reward processing. More specifically, obese children did not show any activity at the amygdala, the parahippocampal gyrus, the thalamus, the striatum (putamen and caudate), the hippocampus, the cingulate cortex, and the orbital frontal cortex. The DLPFC however, was overactive compared to lean individuals. The authors concluded that pronounced frontal lobe engagement suppresses activity of subcortical structures rendering them inefficient in evaluating correctly the rewarding value of food. This leads to a caloric mismatch between consumption and internal body information (Davids et al., 2009). Compared to the current findings, 5‐HTP seems to aid the activation of subcortical structures of reward processing, appraisal, and engagement because activity was observed in the above mentioned regions. Similar results with David's et al. (2009) have also been obtained by Cornier et al. (2009) who compared individuals prone to weight gain (reduced obese) with individuals resistant to weight gain. Both groups were under dietary restraints tailored to each individual's caloric needs. Lean compared to previously obese individuals, while viewing food high in hedonic value showed heightened activity in regions relating to increased feeding motives as well as visual processing. However, when both groups consumed on average 30% more calories than their daily requirements, individuals prone to weight gain when compared to lean individuals, showed increased activity in areas of visual processing and motivation as well as in the hypothalamus. These results suggest that previously obese individuals fail to sense an ability of positive energy balance. In this study, an increased activity was observed at the limbic system and frontal lobes after 5‐HTP consumption. In light of the results presented here as well as the findings of previous studies (Cornier et al., 2009; Davids et al., 2009), it seems that 5‐HTP aids the activation of regions that relate to the evaluation of reward, macronutrient selection, and enteroceptive awareness of internal physiological needs. This is probably one of the primary reasons that studies which prescribe 5‐HTP to obese individuals show significant weight decrease as it aids the activation of structures responsible for external caloric evaluation and internal physiological demands (Cangiano et al., 1992, 1998; Ceci et al., 1989). Moreover Tataranni et al. (1999) observed a negative correlation between insulin levels and the activity of the insular and the orbitofrontal cortex after individuals received a liquid meal replacement formula. Furthermore, a negative correlation was observed between levels of fatty acids and the anterior cingulate cortex, whereas a positive correlation was observed in the DLPFC. These correlations seem to share similarities with the findings of this study. Since insulin and fatty acids are the major transport system of tryptophan, the increased activity observed at the DLPFC, VMPFC, and insula may represent an effort by the organism to maintain a balanced choice between food stimuli and interoceptive needs, maintaining physiological homeostasis.

Extending the analyses beyond the orexigenic network and focusing on the cerebral effects of 5‐HTP intake, results showed maximum activity in the left LG, an area positively coupled with appetite inhibition and satiety in anorexics (Cangiano et al., 1992, 1998; Ceci et al., 1989; Santel et al., 2006). Furthermore, it was postulated by Santel et al. (2006) that the right LG of the occipital lobe shows less activity in anorexics in condition of hunger, whereas increased activity was observed on the left LG in conditions of satiety. Thus, it is believed that in contrast to the left LG, the observed weaker activity of the right LG implies stronger dietary restrain and decreased eating behavior under 5‐HTP intake resembling activation patterns previously found in anorexics. Another area associated with the satiation domain prevalent in this study is the cingulate cortex (Santel et al., 2006; Small et al., 2001). Adding to this Cornier et al. (2009) found significantly stronger activity in the posterior cingulate of thin individuals (resistant to obesity and weight gain) in contrast to obese (prone to weight gain). In line with this latter point, it has been postulated that this limbic structure, due to its central position, has a contributing role in the regulation of body weight as well as thinness. Bold responses have also been recorded on the caudate region as well as the FG. These regions have been associated with the correct appraisal of reward in individuals with normal weight, and according to Davids et al. (2009) failure to activate these regions leads to overeating. In this study, bilateral activity was observed in the DLPFC (BA 44, 47) which has been argued to be involved in the evaluation of tastes as well as oil perception (of any kind) (De Araujo & Rolls, 2004). Furthermore, Cornier et al. (2009) argued that prefrontal lobe activity observed in lean individuals rather than obese, helps them maintain a healthy body weight by inhibiting insular and hypothalamic function. Moreover, although Tataranni et al. (1999) was one of the first who stressed the involvement of the DLPFC and the VMPFC in satiety in other neuroimaging studies shared similar results (Del Parigi et al., 2002; Simmons et al., 2005). In line with previous studies, activity was observed in the right DLPFC (BA 44) as well as VMPFC (BA 4447) bilaterally, a region which has been specifically associated with a rising amount of conflict (Davids et al., 2009). Studies on rodents showed that 80% of the 5‐HT postsynaptic receptors are situated in the frontal lobe, which might account for the observed increased activity of frontal areas (Amargos‐Bosch et al., 2004).

Based on the physiological needs of the organism, the cortex selects those foods that will cover the physiological needs of the organism and avoids others that are redundant. For example, in the case of 5‐HTP, individuals were expected to show regional activity associated with protein stimuli since literature suggests that selection of this food category is affected by 5‐HT levels (Fadda, 2000; Kaye et al., 2009; Morris et al., 1987; Young et al., 1988). In contrast, controls (vitamin C) should be more responsive to high caloric and fatty foods (Carbohydrates & Fats) since not only are they more palatable but also provide the “vessel” (e.g., insulin) for the uptake of additional TRP, crucial for healthy mental function (Comer, 2009; Drewnowski, & Greenwood, 1983). It would be surprising to expect a protein preference in controls since it would decrease levels of 5‐HT and promote LNAAs competition (Fadda, 2000; Weingarten & Elston, 1991; Young et al., 1988). Support for macronutrient selection can be found in studies that examined cortical activity in response to cravings (Pelchat et al., 2004; Small et al., 2001), overconsumption of chocolate despite satiety (Small et al., 2001) as well as incentive motivation and food selection (Arana et al., 2003). Small et al. (2001) observed that the rewarding value of eating chocolate decreased activity in primary gustatory regions of reward processing and feeding motivation. Particularly, these decreases were located in the insula, the caudomedial OFC, and the dorsal striatum. In contrast, during satiety and as the rewarding value of chocolate decreased the following activity was located at motor and premotor areas, the left lateral PFC (middle and inferior frontal gyri), the caudolateral OFC, the right anterior cingulate, and parahippocampal gyrus. Moreover, in the case of Pelchat et al. (2004), it was observed that individuals under monotonous diets activated no frontal cortices but rather the left parahippocampus, the left FG, the right amygdala, the cingulate gyrus bilaterally as well as the right putamen and left caudate nucleus. In addition, comparison between individuals of balance and monotonous diets yielded activation clusters at the left hippocampus and insula as well as the right caudate nucleus. Finally, in another study when individuals were asked to judge from a menu, the appetitive incentive value of food, areas of the amygdala and the medial orbitofrontal cortex were activated (Arana et al., 2003).

The comparison for the 5‐HTP group in favor of food rich in calories and carbohydrates compared to food high in protein content indicated activity bilaterally at the parahippocampal gyrus as well as the right VMPFC (BA 47). These findings, which are in agreement with the study by Small et al. ( 2001) show an effort by the cortex to stop a feeding episode towards a particular food category. The reason behind this event is the fact that physiologic needs in terms of serotonin are covered. No activity was observed in the insula or at the dorsal striatum. In fact the insula/claustrum, the right caudate, the right posterior cingulate gyrus (BA 31), the right thalamus, and the right medial OFC showed increased activity during the contrast in favor of protein rather than high calories and carbohydrates. These regions which are in agreement with the finding by Pelchat et al. (2004), Small et al. (2001), and Tataranni et al. (1999) indicate the initiation of a feeding episode and the motivation to engage with the presented food as it is signified by the activity of the medial OFC (Arana et al., 2003). On the other hand, the vitamin C group showed bilateral activity in response to protein at the anterior cingulate gyrus (BA 24, 31) as well as the left caudolateral OFC (BA 10). Activation of the caudolateral OFC signifies a restraining, engagement effect, toward food rich in protein content and the suppression of limbic structures (Small et al., 2001) as well as a decrease in the rewarding value of the food presented. Although no activity was observed at the cingulate gyrus in response to carbohydrates and high calories, activity was observed on the VLPFC (BA 45) along with parts of the occipital lobe and the right inferior temporal gyrus (BA 37). In the case of Vitamin C group and the high caloric contrast, activity at the lateral PFC may signify a silencing effect of subcortical structures and the initiation of a feeding episode as activation of this region in the absence of other limbic structures has been associated with overeating (Davids et al., 2009). Activity at the FG was also present in both contrasts independent of food content and group. The FG is an area responsible for object recognition, which shows increased activity according to the perception of emotional expression and heightened levels of attention (Kanwisher, McDermott, & Chun, 1997). LaBar et al. (2001) stated that activity of the anterior FG (BA 37) in response to food correlates according to the motivation state of the individual. Furthermore, they added that FG function represents the current reinforcing value of the object to the individual; thus, according to the reward, there is an appropriate BOLD response. In addition, this region along with the hippocampus and amygdala “regulate goal‐oriented behaviors by signaling sensory cues that are relevant to the motivational needs of the organism” (LaBar et al., 2001, p. 495). Finally, bilateral activity was observed for the vitamin C group as well as the 5‐HTP group at the IPL (BA 40) in response to the protein contrast. The IPL is involved in “somatosensory‐gustatory responsiveness” and the reason why this region is active in favor of proteins is probably because both groups are under states of hunger.

Although the results presented here in relation to food preference do incline toward the main hypotheses, an overt confidence cannot be guaranteed, as the topic of food selection according to the value of macronutrients is underdeveloped. Nevertheless, the results of the 5‐HTP group do show a more responsive cortex toward food and food categories, which indicates that some sort of additional cognitive evaluative process is taking place guiding toward a more holistic evaluation of the foods’ macronutrient context.

### 4.1 5‐HTP intake causes recall of food with fewer calories

In line with previous studies, the 5‐HTP group reported less calories than the control group although result did not reach statistical significance. Ceci et al. (1989) reported that obese individuals in the 5‐HTP group, without making any conscious effort to restrict their appetite, consumed on average 500 kcal less than a control group. In addition, Cangiano et al. (1992, 1998) observed that 5‐HTP intake in obese and patients with diabetes was associated with early satiety, a significant reduction in carbohydrate intake as well as weight loss. The obese group, after 5‐HTP intake, lost approximately 2% of their body weight in the first 6 weeks and 3% after 6 weeks. Thus, a woman who takes 5‐HTP and weighs 100 kg, with no diet prescription will lose 5 kg within 3 months of diet. In addition, diabetic individuals treated with 5‐HTP not only lost weight but decreased their overall energy intake of which 75% was accounted to carbohydrates and 25% to fats. In line with the findings by Cangiano et al. (1992, 1998) are the current findings on food recall by the 5‐HTP group in which participants recalled foods with less fat content that controls.

### 4.2 Implications and future directions

Activity was not observed in the hypothalamus. These regions have been reported to be active by PET and fMRI studies in which participants were compared under conditions of hunger and satiety (Fuhrer et al., 2008; Tataranni et al., 1999). Furthermore, only three fMRI studies reported the hypothalamic region to be active, and all of them compared high caloric and low caloric food conditions (Cornier et al., 2009; Laan et al., 2011). However, in the current experimental paradigm, all individuals were tested on an empty stomach (hunger) and stimuli were selected based on their nutritional content, not on their caloric value. It is also of note that the amygdala is susceptible to signal loss (Calvert, Spence, & Stein, 2004).

This research serves as a pilot study for further explorations of 5‐HTP in food selection. Clearly participant numbers are relatively low and restrictive on the inferences that can be drawn. Furthermore, in order to draw direct conclusions on 5‐HT, it would be good to take blood samples measuring serotonin levels prior to, as well as after supplement administration (Wolfe, Metzger, & Jimerson, 1995; Young et al., 1988). In addition, in order to understand how important 5‐HT levels are for weight and appetite control, an inverse model of this study is required. So, for example, a TRP‐free amino acid mixture can be given or a TRP‐free diet can be prescribed safely dropping (temporarily) 5‐HT levels (Dingerkus et al., 2012). Follow‐up studies should take into account hormonal therapies because hormonal imbalance has been associated with weight gain and fluctuation in food intake (Buffenstein, Poppitt, McDevitt, & Prenitce, 1995). In extent, the date of the menstrual cycle should be recorded and taken into account since dietary requirement for females may change during the premenstrual phase as a result of low serotonin activity (Dye & Blundel, 1997). Finally, in order to have greater control over the quantities of food that the individuals recall during the cephalic phase, it would be wise to have a fixed range of choice, for example, restaurant menus from a variety of international cuisines. This would allow individuals to pick the most appropriate meal that they would like to consume at the specific moment; since during the experiment, there were occasions where the individuals referred to normal portions or slices.

## 5 Conclusion

This study has highlighted food‐selective regions as well as illustrated altered patterns of brain responses due to 5‐HTP administration. 5‐HTP aids the activation of regions implicated in feeding behavior. Bold responses were collected from regions implicated in the initiation of a feeding episode (such as the thalamus), the evaluation of reward (such as the caudate, putamen, caudomedial OFC), interoceptive physiological awareness and exteroceptive integration (such as the insula and the hippocampus) as well as behavioral engagement or inhibition (such as the amygdala, the dorsolateral and VMPFC). According to previous studies, individuals who fail to efficiently activate the orexigenic network are either obese or run an increased risk for weight gain. Moreover, the responses of the 5‐HTP compared to the vitamin C group seems to impose greater control over the evaluation and consumption of food as it is implied by the activation of the DLPFC and VMPFC. Clusters of activity in favor of protein for the 5‐HTP group were located in the limbic system and at the basal ganglia, whereas activation toward this particular category for the vitamin C group was located at the anterior cingulate cortex, the IPL bilaterally as well as the caudolateral OFC. The vitamin C group activated frontal structures of satiety and behavioral inhibition, respectively, whereas the 5‐HTP group structures for feeding engagement and cravings. Finally, in the case of the carbohydrates, the 5‐HTP group activated regions related to satiety such as the VMPFC and the parahippocampal gyrus. The vitamin C group on the other hand showed an increased responsiveness in the VLPFC, the occipital and temporal lobe which suggests that in the absence of limbic system activation, an increased engagement and attention toward the arousing value of the stimulus presented.

According to the literature on 5‐HT, this neurotransmitter plays a significant role in healthy mental function (Comer, 2009). Serotonin levels in the brain rise according to foods ingested such as carbohydrates due to the production of insulin. In such occasions, amino acid competition is scarce due to insulin production; thus, more TRP is made available to pass the BBB (Maher et al., 1984; Pardridge & Oldendorf, 1975). If an assumption is made that the brain has internal mechanisms regulating food intake then it will choose the appropriate nutrients/foods, which cover any necessary physiological demands. 5‐HTP intake leads to increased serotonin levels. Thus, the brain under this state is more likely to prefer proteins or even fatty foods. Furthermore, the individual will not be craving sugars or carbohydrates since plenty of serotonin is present in the brain's plasma (Fadda, 2000; Morris et al., 1987; Young et al., 1988). When insulin is absent though, it does not allow glucose to enter the fat cells and convert to fat deposits (Lindberg, Coburn, & Striker, 1984). Thus, people who have been prescribed with 5‐HTP lose weight because they are guided away from the combination of carbohydrates and fats which although tasteful, is the primary reason for weight gain (Volkow & Wise, 2005).

## Conflict of Interest

None declared.
